# Tracking Gut Homeostasis: Key Taxa Transitions and Core Network Hyper-Connectivity as Early Signals of Dysbiosis

**DOI:** 10.3390/biomedicines14071508

**Published:** 2026-07-03

**Authors:** Yi Xu, Chunyan Li, Yiming Zhao, Shibo Lei, Wenyu Yang, Siqi Yao, Kaijuan Wu, Jing Huang, Zheng Yu, Shuijiao Chen

**Affiliations:** 1 Department of Gastroenterology, Xiangya Hospital Central South University, Changsha 410008, China; dieralaxy0617@163.com; 2 Department of Microbiology, Xiangya School of Basic Medical Science, Central South University, Changsha 410078, China; fclichunyan@126.com (C.L.); yimingzhao970904@163.com (Y.Z.); lei2102034@163.com (S.L.); ysqzj1999@163.com (S.Y.); yuzheng@csu.edu.cn (Z.Y.); 3 Department of Parasitology, Xiangya School of Basic Medical Science, Central South University, Changsha 410078, China; 15234435723@163.com (W.Y.); wkjcsu@csu.edu.cn (K.W.); jing_huang@csu.edu.cn (J.H.); 4 National Clinical Research Center for Geriatric Disorders, Xiangya Hospital Central South University, Changsha 410008, China

**Keywords:** gut microbiota, resilience, co-occurrence network, longitudinal analysis

## Abstract

**Background**: Although the gut microbiota is generally recognized to remain relatively stable in healthy individuals, its taxonomic composition still undergoes subtle temporal fluctuations. To systematically characterize these dynamic variations, we adopted “enterotypes” as a macroscopic and practical metric to evaluate the structural dynamics of the intestinal microbial community. **Methods**: We longitudinally recruited a cohort of healthy adults and collected a total of 72 shotgun metagenomic fecal samples across approximately 40 days. All samples underwent metagenomic sequencing, and subjects were grouped by their predominant enterotypes and longitudinal fluctuation patterns. We evaluated the microbial markers and the longitudinal co-occurrence network topologies of different groups to clarify the potential factors causing gut microbial fluctuations. **Results**: Longitudinal tracking revealed that those undergoing persistent alterations in microbial communities exhibited diarrhea symptoms, accompanied by markedly greater variability in gut microbiota. The reduction in *Alistipes shahii* is a potential predictive marker for community instability, exhibiting a cross-validated AUC of 0.824 (95% CI: 0.760–0.888). Furthermore, the co-occurrence network and correlation analysis indicated that fluctuating communities exhibited significantly higher clustering coefficients and denser connectivity among core taxa. Rather than indicating robustness, this dense architecture reflected an increased degree of microbial interdependence within the unstable gut microbial community. **Conclusions**: This preliminary study discovered candidate bacteria taxa that may serve as indicators of disturbances in the gut microbiota. Furthermore, the hyper-connectivity during continuous fluctuations suggested that increased interdependent microbial relationships meant diminished gut resilience. These results offer a new perspective for detecting early signals of dysbiosis and understanding mechanisms underlying stability of gut microbiota.

## 1. Introduction

The state of the gut microbiota plays a crucial role in human health and disease, serving as a critical indicator of gut homeostasis and overall well-being. In an ecological context, gut homeostasis is largely assessed by two fundamental properties: resistance (the ability to endure external disturbances) and resilience (the capacity to revert to the original baseline after a disruption). Microbial communities devoid of resistance and resilience are prone to transitioning into dysbiosis [1]. Longitudinal studies [2,3] demonstrated that patients with inflammatory bowel disease (IBD) and irritable bowel syndrome (IBS) displayed less temporal stability in their gut microbiota relative to healthy individuals. Our previous work [4] also showed variation within healthy adults. Each person showed a different ability to resist external disturbances. This resistance often correlates with enrichment of certain probiotic taxa. Nevertheless, it is crucial to acknowledge that homeostasis is not a rigid, static condition, but a highly individualized dynamic equilibrium [5]. Homeostasis functions as a personalized and dynamic balance. Even stable adults show small and continuous shifts in gut microbial composition [6]. Although this conceptual paradigm is widely acknowledged, the definitive boundary distinguishing benign homeostatic fluctuations from dysbiotic, pathological volatility remains largely undefined.

Gut microbiota datasets contain high-dimensional information, including hundreds of species and millions of genes, and also show strong variation between individuals. Analysis based on a large number of microbial taxa often generated “noise”, leading to miss important microbial signals. Therefore, researchers introduced the concept of enterotypes to capture the broad structure of the microbial community [7,8]. Enterotype analysis reduces many dimensions into several key macrostates [9], filters out weak fluctuations of low-abundance bacteria so that provide a reliable indicator for longitudinal tracking. By monitoring the evolutionary trajectory of an individual’s enterotype over time, we can ascertain whether variations in the gut microbiota are benign and dynamic (indicated by the sustained maintenance of the gut type) or whether the stable network has deteriorated and is commencing pathological structural reorganization (evidenced by frequent and intense transitions between different gut types).

Evaluating gut microbiota homeostasis exclusively by abundance-based dimensionality reduction is inadequate. The variability in intestinal homeostasis among individuals experiencing comparable external disturbances indicates that inherent community-level interactions, rather than merely the existence of particular taxa, are pivotal in determining gut resilience [4]. Gut microbes engage in complex ecological networks, involving metabolic cross-feeding, synergistic co-occurrence, and competitive exclusion. Representatively, *Faecalibacterium prausnitzii*, an essential butyrate-producing bacterium, facilitated gut homeostasis by forming metabolic networks with other microorganisms [10,11,12]. *Bacteroides* spp. and *Prevotella* spp., which dominate significant enterotypes, often display mutually exclusive patterns associated with host lipid metabolism [7,13,14,15,16,17]. Thus, the topological characteristics of microbial co-occurrence networks may provide a more reliable measure for assessing homeostasis compared to conventional single-taxon biomarkers.

This research entailed a high-frequency longitudinal pilot experiment to investigate the microbial fingerprints linked to gut homeostasis. Through rigorous sampling, we observed notable interpersonal differences in enterotype stability and determined that *Alistipes shahii* may serve as a potential biomarker for predicting community instability. Our findings revealed that microbiota in disturbed states exhibited more densely interconnected and intricate core networks. This unexpected result challenges the conventional belief that “complexity equals stability”, suggesting instead that hyper-connected networks may indicate compensatory reconfiguration or systemic fragility under stress. This exploratory study provides initial insights into the mechanisms regulating microbiome resilience and proposes a hypothesis-generating framework for evaluating gut homeostasis.

## 2. Materials and Methods

### 2.1. Study Design and Stool Sample Collection

We executed a meticulously controlled longitudinal pilot study with 8 healthy subjects recruited from Central South University. To reduce extrinsic confounding variables, all subjects lived in the same campus setting and adhered to a consistent food regimen. Participants who had utilized antibiotics in the three months preceding sampling were excluded. The consistency of dietary consumption was evaluated by a self-reported questionnaire, where participants indicated any changes in their perceived meat-to-vegetable intake ratio (1 = generally the same, 5 = radically different) over the sample period; individuals with a score exceeding 2 were removed. All the participants resided and conducted daily activities within the same campus. Although these metrics do not eradicate all potential confounders, they were instituted to augment internal validity within the limitations of this pilot investigation. Ultimately, a total of 8 healthy participants meeting the inclusion and exclusion criteria were recruited. Detailed participant demographics, including age, sex, and body mass index (BMI), are provided in Supplementary Table S1. This study was approved by the Institutional Review Board of the Basic Medical College of Central South University (No. 2021-KT75).

Sampling was conducted at two high-frequency phases: an initial baseline week and 1–2 weeks following an unplanned vacation break. Participants self-collected fecal samples every 2–3 days (three samples per week), yielding a total of 72 samples. Prior to storage, the consistency of the stool samples was evaluated via BSS, which ranges from grade 1 (normal, hard stool) to grade 7 (loose, diarrheal stool). A score of ≥6 was defined as diarrhea.

### 2.2. Metagenomic Sequencing and Taxonomic Profiling

Total microbial genomic DNA was extracted from intestinal samples via the E.Z.N.A.^®^ Soil DNA Kit (Omega Bio-Tek, Norcross, GA, USA). Sequencing libraries (approx. 400 bp insert size) were constructed using the NEXTFLEX Rapid DNA Sequencing Kit (BIOO Scientific, Austin, TX, USA) and sequenced on the Illumina NovaSeq 6000 PE150 platform (Shanghai Biozeron Biotech. Co., Ltd., Shanghai, China).

Each sample generated approximately 10 Gb of raw sequencing data. For quality control, raw reads were processed using fastp (v.0.23.4) [18] with a minimum Phred quality score of ≥20, and a maximum N content of 10%, followed by deduplication with FastUniq (v.1.1.0) [19]. Host DNA contamination was removed by aligning reads to the human reference genome (hg38) using Bowtie2 (v.2.5.2) [20], retaining only uniquely mapped reads with the sensitive mode. Taxonomic classification and abundance estimation of the remaining clean reads were performed using Kraken2 (v.2.1.3) with a custom database [21] and Bracken (v.2.9) [22], respectively. To further mitigate the effects of potential cross-sample contamination and sequencing errors, we retained only taxa that were present in at least two biological samples, with special consideration given to those exhibiting a total relative abundance > 0.001%.

### 2.3. Microbiota Analysis and Visualization

Enterotype analysis was primarily conducted via the “*ade4*” [23] and “*cluster*” packages in R (v.4.3.3). Following the methodology of Arumugam et al. [7], we calculated the Jensen–Shannon distances (JSDs) and clustered the samples using the Partitioning Around Medoids (PAM) algorithm. Microbial diversity analyses (Shannon index and Bray–Curtis dissimilarity) were performed using the “*vegan*” package [24], with beta-diversity significance assessed via Permutational Multivariate Analysis of Variance (PERMANOVA). For Linear discriminant analysis effect size (LEfSe), *p*-values were adjusted for multiple comparisons using the Benjamini–Hochberg procedure (false discovery rate < 0.05), and taxa with an LDA score > 4 were considered significantly differential [25]. A random forest [26] classifier was constructed utilizing the “*randomForest*” program to assess the prediction efficacy of candidate biomarkers for identifying community states (stable versus unstable). To alleviate overfitting and evaluate generalizability, we utilized a Leave-One-Subject-Out Cross-Validation (LOSO-CV) approach [27], in which each subject’s longitudinal samples were systematically excluded as the test set. The model was constructed with 500 trees, and class imbalance was mitigated with inverse-proportion weights (“classwt”). Receiver operating characteristic (ROC) curves were plotted using the “*pROC*” package [28], with automatic directionality optimization (direction = “auto”) to correctly capture both positive and negative correlations between taxon abundance and instability. The area under the curve (AUC) and its 95% confidence interval (95% CI) were calculated to assess and compare the discriminative ability of each feature set. To infer putative community interactions, temporal co-occurrence networks were constructed using the “*microeco*” and “*meconetcomp*” packages [29]. To control for false discovery due to multiple hypothesis testing and retain robust correlations, we applied the Benjamini–Hochberg procedure (FDR < 0.05) to adjust the correlation *p*-values. Only correlations meeting both the adjusted significance threshold (FDR < 0.05) and the absolute correlation coefficient threshold (|r| > 0.6) were retained for network visualization and subsequent topological analyses. Network modularity was evaluated using a fast-greedy algorithm via the “*igraph*” package, and network topologies were visualized in Gephi (v.0.10.1). For highly abundant taxa (mean relative abundance > 1%), pairwise Pearson correlations were calculated to characterize specific synergistic or antagonistic relationships. General data visualizations were generated using “*ggplot2*” and “*circlize*” [30] in R.

### 2.4. Other Statistical Analyses

Statistical comparisons of continuous variables were performed using Student’s *t*-test or the Wilcoxon rank-sum test, depending on data normality. Categorical variables were compared using the Chi-square test. Statistical significance was defined as *p* < 0.05.

## 3. Results

### 3.1. Identification and Time-Dependent Stability Assessment

**Figure 1:**
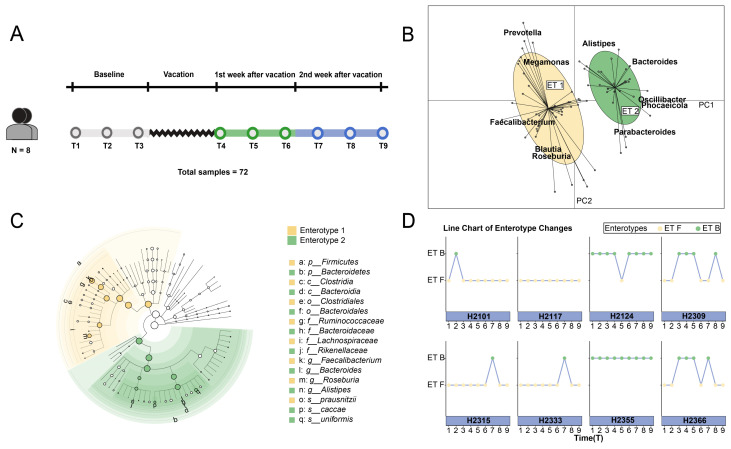
Sample collection and enterotype identification. (**A**) An overview of the entire process of sample collection. T1–9 represent 9 collection points, and “vacation” refers to the interval time between two measurement periods. (**B**) The results of enterotype analysis are visualized via two-dimensional plots following dimensionality reduction via principal coordinate analysis (PCoA). (**C**) LEfSe analysis results showing the differential microbiota between the two groups, which represent two distinct enterotypes. (**D**) Temporal dynamics of enterotypes in 8 subjects.

We longitudinally profiled 72 fecal samples from 8 healthy participants across two high-frequency sampling phases (Figure 1A). Based on Jensen–Shannon distances, PAM clustering stratified the samples into two primary enterotypes (Supplementary Figure S1A,B). Principal coordinate analysis (PCoA) and taxonomic composition assessment (Figure 1B,C) identified Enterotype 2 as a *Bacteroides*-dominated enterotype (ETB). Conversely, Enterotype 1 was distinctly enriched in Firmicutes, particularly *Faecalibacterium* (Supplementary Figure S1C–F), and was thus defined as a *Faecalibacterium*-dominated enterotype (ETF). Crucially, participant-level temporal tracking (Figure 1D) revealed distinct stability profiles. While six individuals maintained highly stable enterotypes throughout the two-month period (classified into the ETF and ETB groups), two individuals exhibited continuous, dynamic enterotype transitions, along with diarrhea that was recorded ≥ 2 times (Supplementary Table S1). These two individuals were categorized as the “Change” group for subsequent comparative analyses.

### 3.2. Diversity and Differences in the Gut Microbial Communities

**Figure 2:**
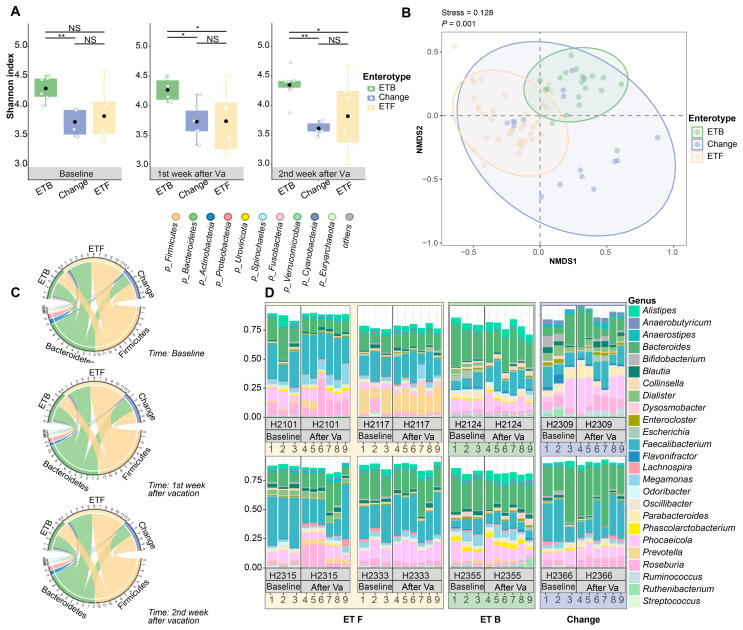
Diversity and differences in the gut microbial communities among the three enterotype groups. (**A**) Comparison of Shannon indices (* *p* < 0.05, ** *p* < 0.01) among the three groups across different time points (baseline, first week after vacation, second week after vacation). (**B**) Results of NMDS analysis, where a stress value < 0.2 indicates a good model fit, and *p* values were calculated via PERMANOVA. (**C**) Chord diagram illustrating the taxonomic composition at the phylum level across the 3 groups at the three time points, with the legend in the upper right corner indicating the top 10 most abundant taxa. (**D**) shows the dynamic changes in the gut microbiota composition at the genus level for each subject, featuring only the 25 most abundant genera.

We conducted α-diversity and β-diversity analyses on samples from three distinct groups. While the longitudinal alpha-diversity (Shannon index) remained relatively stable within individuals (Supplementary Figure S2A), the ETB group exhibited a generally higher overall alpha-diversity compared to the ETF and Change groups (Figure 2A). Notably, non-metric multidimensional scaling (NMDS) of beta-diversity (Figure 2B) demonstrated that the Change group possessed a significantly larger confidence ellipse. This indicates heightened community dispersion and greater structural variability compared to the stable groups. PERMANOVA confirmed significant heterogeneity in community composition among the three groups (Supplementary Figure S2B). Significant heterogeneity in microbial composition was observed among the three groups at both the phylum and genus levels. At the phylum level, mild differences were observed in the taxonomic composition among the three groups (Figure 2C). At the genus level, more significant differences in the temporal fluctuations of the gut microbiota were observed. Similarly, the Change group presented more distinct temporal variations than the other two groups did (Figure 2D).

### 3.3. Screening Sensitive Markers Related to Gut Microbial Fluctuations

**Figure 3:**
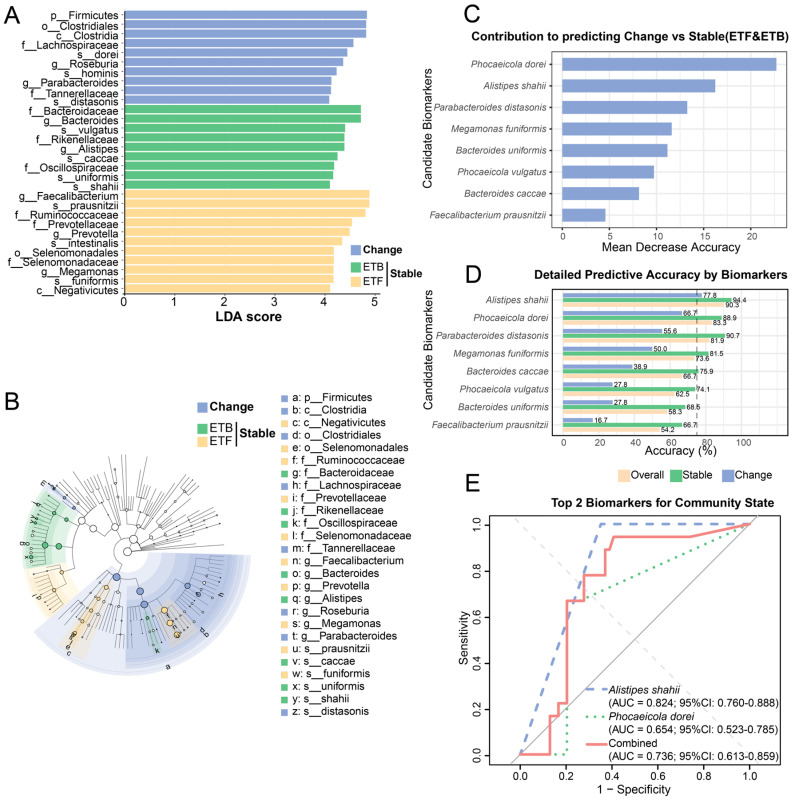
(**A**,**B**) Screening of candidate differential microbial markers. The horizontal histogram (**A**) and branch evolutionary tree diagram (**B**) illustrate the statistically significant microbial biomarkers from the phylum to species level among the three groups, with the horizontal histogram highlighting the top 30 taxa on the basis of their LDA scores (LDA > 4). (**C**) Feature importance ranking derived from the Random Forest model. The bar plot illustrates the relative contribution of the top differential taxa in discriminating between Stable (ETB and ETF) and fluctuating (Change) states of microbial community. The *x*-axis represents the Mean Decrease Accuracy, where a higher value indicates a greater loss in the model’s classification capability if that specific taxon is permuted. (**D**) Detailed classification accuracy of individual candidate biomarkers. This panel disaggregates the predictive accuracy (%) when each taxon is utilized as a standalone feature in the Random Forest model. Colors denote different performance metrics: overall accuracy (gray), accuracy for correctly identifying stable instances (green), and accuracy for detecting fluctuating instances (red). This detailed breakdown highlights the true discriminatory sensitivity of each taxon, particularly in addressing potential class imbalance. (**E**) Receiver Operating Characteristic (ROC) curves comparing the cross-validated predictive performance of three feature sets under the LOSO-CV framework. The curves plot the true positive rate against the false positive rate for *Alistipes shahii* alone (AUC = 0.824, 95% CI: 0.760–0.888), *Phocaeicola dorei* alone (AUC = 0.654, 95% CI: 0.523–0.785), and their combination (AUC = 0.736, 95% CI: 0.613–0.859). The combined panel did not outperform the single *Alistipes shahii* marker, suggesting that the depletion of this key commensal is a more reliable indicator of gut microbial instability.

**Figure 4:**
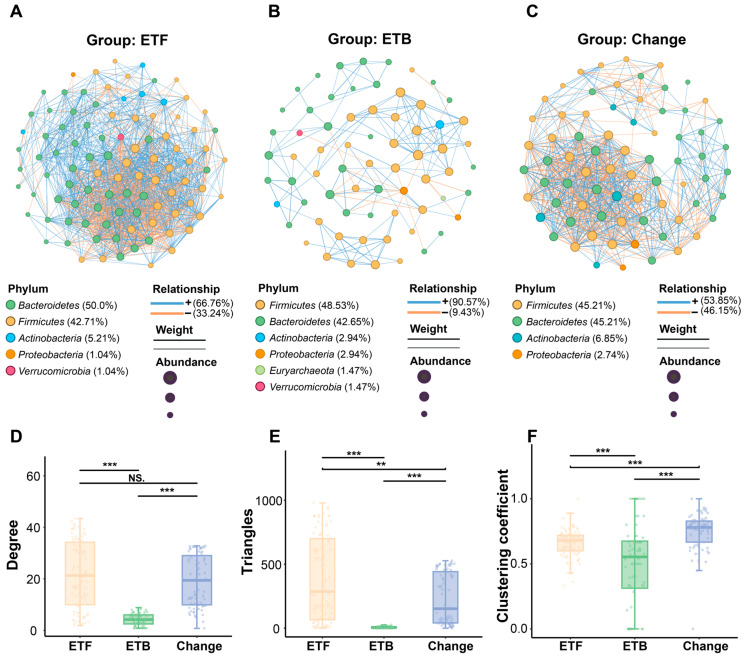
Cooccurrence network analysis of the microbial communities of the ETF, ETB, and Change groups. Network of the ETF (**A**), ETB (**B**), and Change (**C**) groups. All networks are constructed on the basis of the same parameters of an absolute correlation coefficient *R* > 0.6 and a *p* value < 0.001. Comparisons of network parameter degrees (**D**), triangles (**E**), and clustering coefficients (**F**) among the three groups (NS, no significant difference; **, *p* < 0.01; ***, *p* < 0.001); higher values suggest greater complexity of the microbial networks.

To explore the core species associated with perturbations in the gut microbiota, we initially employed LEfSe to identify differentially enriched taxa (Figure 3A,B). At the genus level, the ETF group was significantly enriched in *Faecalibacterium*, *Prevotella*, and *Megamonas*; the ETB group was significantly enriched in *Bacteroides* and *Alistipes*; and the Change group was significantly enriched in *Roseburia* and *Parabacteroides*. Subsequently, eight candidate species—which were significantly enriched at the species level and derived from the aforementioned differential genera—were prioritized to construct a Random Forest (RF) classification model to evaluate their predictive potential for community stability. Based on the Mean Decrease Accuracy (MDA) shown in Figure 3C, *Phocaeicola dorei* emerged as the most contributory feature, followed by *Alistipes shahii* and *Parabacteroides distasonis*. This ranking highlighted their crucial function as sentinel indicators for alterations in gut homeostasis. Figure 4D demonstrates the results of predictive efficacy of the chosen biomarkers for “stable” (ETB and ETF) and Change group, respectively. *Alistipes shahii* achieved an overall accuracy of 90.3%, demonstrating remarkable sensitivity in differentiating stable (94.4% versus 75%) and unstable (77.8% versus 25%) phases. Likewise, *Phocaeicola dorei* demonstrated strong performance with an overall accuracy of 83.3%. Therefore, ROC curve analysis based on the stringent LOSO-CV was conducted to compare the predictive performance of aforementioned taxa (Figure 3E). *Alistipes shahii* alone achieved an AUC of 0.824 (95% CI: 0.760–0.888). In comparison, *Phocaeicola dorei* alone yielded an AUC of 0.654 (95% CI: 0.523–0.785), while the combined two-taxon panel produced an AUC of 0.736 (95% CI: 0.613–0.859). These data indicate that when monitoring the early disruptions of intestinal flora homeostasis, the reduction in *Alistipes shahii* may be the most promising indicator species.

### 3.4. Network Co-Occurrence and Correlation Analysis of the Gut Microbiota Among Various Enterotypes

We further inquired if the structural configuration of microbial co-occurrence networks varied among the three community states. Employing rigorous thresholds, we constructed networks for the ETF, ETB, and Change groups (Figure 4A–C). As shown in Supplementary Table S2, the Change group generated a comparatively little network with 73 nodes, but it contained more than four times as many edges (793) as the ETB group., which had 68 nodes and 159 edges. The ETF group exhibited the highest total connectedness (96 nodes, 1435 edges). Topological analyses demonstrated a distinct pattern (Figure 4D–F). The node degree and triangle counts were significantly higher in both the ETF and Change groups compared to the ETB group (*p* < 0.001). While Change group demonstrated a considerably higher clustering coefficient than both stable groups—indicating that its microbial connections were not only numerous but also structured into strongly related cliques. This form of hyper-connectivity does not inherently indicate a robust ecosystem; instead, it may signify a condition in which essential taxa are compelled into more intense positive interactions.

**Figure 5:**
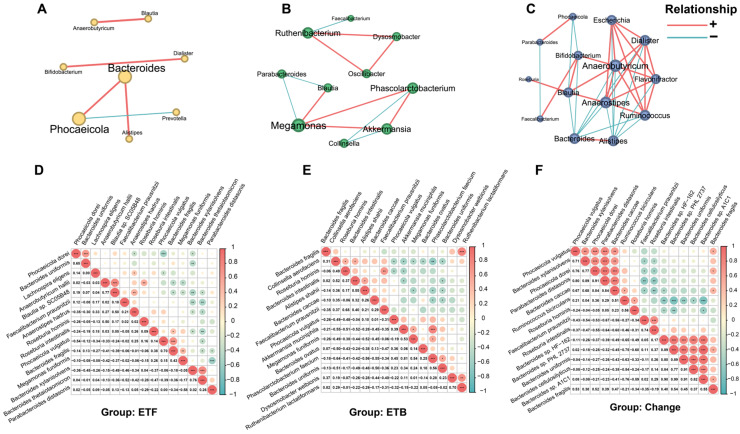
Correlation analysis among the ETF, ETB, and Change groups. (**A**–**C**) Figure (**A**), (**B**), and (**C**) depict the network topologies for the ETF, ETB, and Change groups, respectively. The networks were constructed utilizing the top 15 most abundant bacterial genera within each group. The edges connecting the nodes denote strong and statistically significant Spearman’s rank correlations (∣R∣ > 0.6, *p* < 0.05). Red edges indicate positive correlations, whereas blue edges signify negative correlations. (**D**–**F**) Species correlation heatmaps of the 15 most abundant species in the ETF, ETB and Change groups. (*, *p* < 0.05; **, *p* < 0.01; ***, *p* < 0.001).

We further analyzed whether the core taxa—the 15 most dominant genera and species—exhibited variations in their relationship patterns among the three groups (Figure 5). At the genus level, the stable ETF and ETB groups had a significant characteristic: their core co-occurrence networks were considerably sparse in contrast to the Change group, which displayed both more frequent and stronger connections (Figure 5A–C). This disparity continued at the species level. The heatmaps revealed that the Change group had a complex network of both positive and negative correlations, while the stable groups presented only sparse, primarily weaker connections (Figure 5D–F). Collectively, these findings indicate that community disturbance is associated not only with a change in taxonomic composition but also with a reconfiguration of the core interactome, wherein formerly weak or nonexistent relationships become more closely interconnected.

## 4. Discussion

Consistent with our core hypothesis regarding gut homeostatic dynamics, this 40-day high-frequency longitudinal study provided direct evidence linking enterotype instability, microbial network shifts and early gut dysbiosis signals in healthy individuals. Our findings indicated that individuals undergoing persistent enterotype transitions also encountered numerous diarrhea episodes, and their longitudinal community variation—evidenced by a considerably larger NMDS confidence ellipse and increased within-group Bray–Curtis distances—was significantly greater than that of the stable groups (ETB and ETF groups). Furthermore, *Alistipes shahii* may serve as a possible biomarker for predicting gut microbiota instability. Co-occurrence network research revealed that disrupted microbiota possessed a more intricately integrated and complicated core functional network, indicating that excessive microbial coupling within the core community may paradoxically diminish ecosystem resilience.

To mitigate confounding variables related to dietary or lifestyle changes, we recruited the sample using stringent screening criteria. Nonetheless, two of the eight participants underwent recurrent enterotype transitions throughout the sampling period, which correlated with several self-reported instances of diarrhea. Diarrhea and gut microbial disruption are known to mutually promote one other [31,32,33]. Thus, their co-occurrence indicates true ecological instability rather than mere benign fluctuation. Consistent with this, NMDS indicated that the temporal dispersion of the Change group was significantly greater than that of the ETF and ETB groups, underscoring that these longitudinal variations were improbable to arise from random sampling noise. The stability of an individual’s enterotype serves as a straightforward yet insightful indicator of the gut ecosystem’s integrity.

We subsequently examined the microbiological factors linked to these ecological disturbances. Both LEfSe studies and machine learning models consistently identified *Alistipes shahii* as the most reliable predictor of microbial instability. Within the rigorous LOSO-CV framework, *Alistipes shahii* attained an AUC of 0.824, surpassing both *Phocaeicola dorei* individually (AUC = 0.654) and their combined performance (AUC = 0.736). This unforeseen discovery—that the inclusion of *Phocaeicola dorei* did not enhance predictive performance—indicates that the two species do not function synergistically; instead, the signal from “*Phocaeicola dorei*” may generate inter-individual variability that obscures the more predictable reduction in *Alistipes shahii*. Significantly, whereas both species are prevalent residents of the gut, they were not commonly acknowledged as probiotics or pathogens. Their protective or harmful roles seemed to be significantly influenced by the circumstances, particularly the host’s health condition and intestine ecological environment. An animal study [34] indicated that oral administration of the *Alistipes shahii* As360 strain mitigated colitis symptoms, implying a protective capacity against diarrhea-related intestinal inflammation. Interestingly, a heightened presence of Alistipes shahii has been noted in conjunction with intestinal inflammation and histopathological damage in specific experimental models [35], while its levels were markedly reduced in healthy individuals adhering to plant-based diets [36]. These contradictory observations preclude a definitive conclusion as to whether the decline in *Alistipes shahii* abundance represents a cause or a consequence of gut microbial disturbance. Nevertheless, regardless of its causal direction, we propose that the depletion of this taxon should be regarded as a sensitive candidate microbial indicator of community stress. Concerning *Phocaeicola dorei* (previously classified as *Bacteroides dorei*), one investigation demonstrated its capacity to alleviate DSS-induced experimental colitis through the modulation of bile acid metabolism [37], whereas another study [38] indicated a significant enrichment of this species in individuals with diarrhea-predominant irritable bowel syndrome. Considering the contradictory findings and our observation that *Phocaeicola dorei* did not augment predictive capability, we suggest that its proliferation is probably a secondary occurrence rather than a primary catalyst of instability. The significant inter-individual variability in bloom timing and size may elucidate why its inclusion generates noise rather than synergy in cross-validation contexts. Our findings collectively suggested that the reduction in *Alistipes shahii* served as the most dependable early warning indicator of gut community disruption. The moderate cross-validated AUC of 0.824 emphasizes the intrinsic inter-individual variability in gut microbial dynamics and reinforces the essential requirement for validation in larger, independent cohorts.

The network co-occurrence analysis elucidated the ecological process underlying the disturbance. Network co-occurrence analysis encompassing all species indicated that the total complexity of the Change group surpassed that of the ETF and ETB groups; yet, the ETF group demonstrated much greater complexity than the ETB group. This indicated that overall network complexity, in isolation, was an inadequate differentiator of community stability—a conclusion that contradicts the traditional assumption that more interconnected ecosystems are intrinsically more resilient [39]. Recent research on the gut microbiota have challenged this generalization: increased complexity may correlate with diminished resilience and potential ecological collapse, especially when positive interactions prevail [40]. Long-term unbalanced diets have been demonstrated to enhance overall network complexity and positive interactions; nevertheless, this structure renders the microbiota less resilient to disturbances such as antibiotics, indicating reduced stability [41]. This transpires due to the presence of intricate networks that often contain significant positive interactions (cooperation and mutualism), establishing critical tipping points where the removal of a single keystone node can precipitate abrupt network failure [42]. Authoritatively, the study of Corral López et al. [43] highlighted that in healthy settings, gut microbial networks were mostly influenced by competitive interactions, while dysbiosis was linked to an increasing dominance of positive interactions and a reduction in negative interactions. Our observation that the ETF and ETB groups demonstrated a markedly greater proportion of positive correlations compared to the Change group seems to contradict this hypothesis. However, the comprehensive co-occurrence network encompasses the complete range of community members, including low-abundance species that do not have essential metabolic roles. These species may function as background noise, obscuring the authentic topological characteristics of the core interactome. Significantly, when we focused on the 15 most prevalent taxa at both the genus and species levels, positive interactions emerged as the predominant influence in the Change group. This suggests that the fundamental microbial network of a disrupted community is marked by excessive positive connection, aligning with the idea that hyper-connected cooperative relationships at the core signify dysbiosis. These discoveries offer methodological direction for more precisely delineating the authentic topological characteristics of gut microbial networks: a core-taxon-focused study can unveil key microbial patterns that are otherwise concealed by noise from low-abundance members. Although enterotype-based stratification offers a pragmatic framework for longitudinal monitoring, it is important to recognize that it constitutes a heuristic simplification of continuous compositional gradients and may be influenced by the selection of clustering algorithms and sample composition [8]. The co-occurrence networks presented are based on correlation studies and should be regarded as hypothesis-generating representations of potential microbial connections, rather than as conclusive proof of direct ecological interactions.

There are some limitations in this exploratory pilot study. First, the sample size was limited (N = 8), and individuals were sourced from a single university campus exhibiting a homogeneous food pattern. This controlled environment reduced confounding variables but consequently limits the generalizability of our results. Moreover, although we accounted for significant dietary and environmental confounders via rigorous recruiting criteria, we did not routinely gather data on individual-level variations in stress, sleep quality, or other lifestyle factors during the holiday period. These unmeasured factors may have associated with microbiota variation [44] and should be addressed in future studies. Secondly, the “Change” group comprised only two individuals, limiting statistical power to detect synergistic effects between taxa. Despite the implementation of a rigorous LOSO-CV to reduce overfitting, the finding that the combined two-taxon panel did not exceed the performance of the single marker may be somewhat ascribed to the constrained sample size. This constrains the statistical power to detect genuine synergistic interactions between the two species. Larger cohorts with sufficient numbers of unstable individuals would be better equipped to determine whether *Alistipes shahii* and *Phocaeicola dorei* engage in true ecological interactions or merely co-occur as independent responses to community stress. Therefore, our analysis relied on taxonomic profiling alone; the absence of metabolomic, transcriptomic, or host inflammatory data limits our ability to elucidate functional mechanisms and host–microbe interactions. Ultimately, as an observational study, we identified correlations rather than conclusive causative links between the dynamics of *Alistipes shahii* and intestinal disturbances. The role of the observed microbial bloom or depletion as either a primary cause or a secondary responder to gut dysbiosis has yet to be clarified. Future extensive longitudinal cohorts and mechanistic experiments (e.g., mice models) are necessary to confirm these candidate biomarkers and investigate their particular ecological functions in preserving gut homeostasis. What we wish to emphasize is that the present study is an exploratory and preliminary study. Our findings are not intended to provide solid recommendations or definitive conclusions. Instead, this work serves to generate hypotheses and offer early signals that may inform the design of future, larger-scale validation studies.

## 5. Conclusions

This pilot longitudinal investigation demonstrated that enterotype stability can function as a macroscopic indication of gut homeostasis. We discovered that *Alistipes shahii* serves as a promising signal of gut microbial instability, reflecting community disturbances not as a causative agent but as a sensitive ecological indicator. Co-occurrence network analysis revealed that global network complexity could not differentiate between stable and unstable communities; rather, hyper-connectivity among key taxa characterized the perturbed state. These findings collectively suggest novel avenues for future research on the early detection of dysbiotic signals and provide distinct insights into the microbial traits associated with disruptions in gut microbiota homeostasis.

## Data Availability

The raw sequence data reported in this paper have been deposited in the Genome Sequence Archive [45] of the National Genomics Data Center [46], China National Center for Bioinformation/Beijing Institute of Genomics, Chinese Academy of Sciences (GSA: CRA025868), which is publicly accessible at https://ngdc.cncb.ac.cn/gsa (accessed on 12 August 2025).

## Supplementary Materials

The following supporting information can be downloaded at: https://www.mdpi.com/article/10.3390/biomedicines14071508/s1. Supplementary Figure S1: Clustering of enterotypes and identification of representative microbiota; Supplementary Figure S2: The results of the microbial community analysis for the ET F, ET B, and Change groups; Table S1: Metadata information for all volunteers; Table S2: The co-occurrence analysis parameters of microbial networks in the three groups.

## Abbreviations

PAM	Partitioning Around Medoids.
C–H index	Calinski–Harabasz index.
LEfSe	Linear discriminant analysis effect size.
LOSO-CV	Leave-One-Subject-Out Cross-Validation.
FDR	False discovery rate.
SCFAs	Short-chain fatty acids.
BSS	Bristol stool form scale.
PCoA	Principal coordinate analysis.
NMDS	Nonmetric multidimensional scaling.
ETB	*Bacteroides* enterotype.
ETF	*Faecalibacterium* enterotype.
