# Supplementary material for: Tracking Gut Homeostasis: Key Taxa Transitions and Core Network Hyper-Connectivity as Early Signals of Dysbiosis
Source: Biomedicines. 2026 Jul 3;14(7):1508. doi: 10.3390/biomedicines14071508 (PMC13404127; doi:10.3390/biomedicines14071508)
Supplement: Supplementary file 1 [file biomedicines-14-01508-s001.zip › Supplementary Figure S1.pdf]

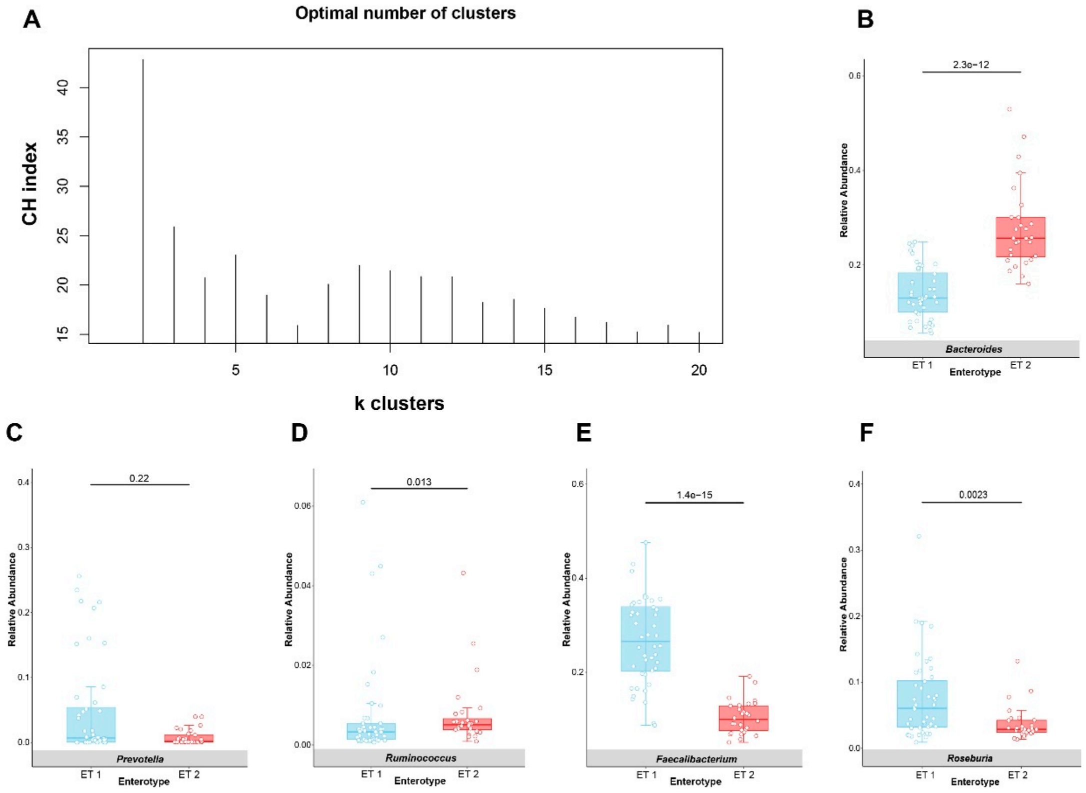

Abbreviation: ET 1, enterotype 1; ET 2, enterotype 2

Supplementary Figure S1: Clustering of enterotypes and identification of representative microbiota. (A) illustrates the optimal number of clusters for the 72 samples. (B-F) depict the comparative results of the relative abundance of several representative bacterial genera between the two enterotypes from the clustering analysis.
