# Supplementary material for: Tracking Gut Homeostasis: Key Taxa Transitions and Core Network Hyper-Connectivity as Early Signals of Dysbiosis
Source: Biomedicines. 2026 Jul 3;14(7):1508. doi: 10.3390/biomedicines14071508 (PMC13404127; doi:10.3390/biomedicines14071508)
Supplement: Supplementary file 1 [file biomedicines-14-01508-s001.zip › Supplementary Figure S2.pdf]

A

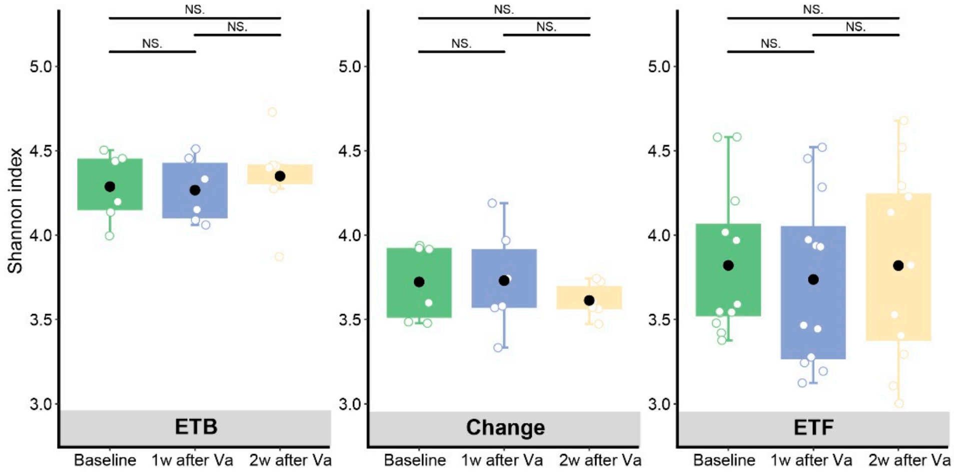

B

pairs	R2	p.value	p.adjusted
ETF vs ETB	0.2574506	0.001	0.001
ETF vs change	0.1532996	0.001	0.001
ETB vs change	0.2486935	0.001	0.001

Abbreviation: 1w after Va, first week after vacation; 2w after Va, second week after vacation.

Supplementary Figure S2: The results of the microbial community analysis for the ET F, ET B, and Change groups. (A) showed the comparison of the Shannon index among the three groups at different sampling time points. (B) depicted the pairwise comparison results among the three groups based on PERMANOVA analysis.
