# Supplementary material for: Tracking Gut Homeostasis: Key Taxa Transitions and Core Network Hyper-Connectivity as Early Signals of Dysbiosis
Source: Biomedicines. 2026 Jul 3;14(7):1508. doi: 10.3390/biomedicines14071508 (PMC13404127; doi:10.3390/biomedicines14071508)
Supplement: Supplementary file 1 [file biomedicines-14-01508-s001.zip › Supplementary Table S1.pdf]

Table S1 **Metadata information for all volunteers**

ID	People	Gender	Age	BMI	blood type	Antibiotics	Diet change	BSS grade (only grade≥6 was recorded)
H2101A	H2101	Female	26	20.83	A	NO	2	
H2101B	H2101					NO		
H2101C	H2101					NO		
H2101D	H2101					NO		
H2101E	H2101					NO		
H2101F	H2101					NO		
H2101G	H2101					NO		
H2101H	H2101					NO		
H2101I	H2101					NO		
H2117A	H2117	Female	24	21.75	B	NO	0	
H2117B	H2117					NO		
H2117C	H2117					NO		
H2117D	H2117					NO		
H2117E	H2117					NO		
H2117F	H2117					NO		
H2117G	H2117					NO		
H2117H	H2117					NO		
H2117I	H2117					NO		
H2124A	H2124	Female	23	21.8	O	NO	2	
H2124B	H2124					NO		
H2124C	H2124					NO		
H2124D	H2124					NO		
H2124E	H2124					NO		
H2124F	H2124					NO		
H2124G	H2124					NO		
H2124H	H2124					NO		
H2124I	H2124					NO		
H2309A	H2309	Female	23	18.87	AB	NO	2	
H2309B	H2309					NO		
H2309C	H2309					NO		
H2309D	H2309					NO		
H2309E	H2309					NO		
H2309F	H2309					NO		
H2309G	H2309					NO		
H2309H	H2309					NO		6
H2309I	H2309					NO		6
H2315A	H2315	Female	23	20.2	A	NO	0	
H2315B	H2315					NO		
H2315C	H2315					NO		
H2315D	H2315					NO		

H2315E	H2315					NO		
H2315F	H2315					NO		
H2315G	H2315					NO		
H2315H	H2315					NO		
H2315I	H2315					NO		
H2333A	H2333	Female	23	28.93	B	NO	2	
H2333B	H2333					NO		
H2333C	H2333					NO		
H2333D	H2333					NO		
H2333E	H2333					NO		
H2333F	H2333					NO		
H2333G	H2333					NO		
H2333H	H2333					NO		
H2333I	H2333					NO		
H2355A	H2355	Female	25	20.4	B	NO	1	
H2355B	H2355					NO		
H2355C	H2355					NO		
H2355D	H2355					NO		
H2355E	H2355					NO		
H2355F	H2355					NO		
H2355G	H2355					NO		
H2355H	H2355					NO		
H2355I	H2355					NO		
H2366A	H2366	Male	24	21.68	NA	NO	2	6
H2366B	H2366					NO		
H2366C	H2366					NO		
H2366D	H2366					NO		6
H2366E	H2366					NO		6
H2366F	H2366					NO		
H2366G	H2366					NO		
H2366H	H2366					NO		
H2366I	H2366					NO		
