# Supplementary material for: Tracking Gut Homeostasis: Key Taxa Transitions and Core Network Hyper-Connectivity as Early Signals of Dysbiosis
Source: Biomedicines. 2026 Jul 3;14(7):1508. doi: 10.3390/biomedicines14071508 (PMC13404127; doi:10.3390/biomedicines14071508)
Supplement: Supplementary file 1 [file biomedicines-14-01508-s001.zip › Supplementary Table S2.pdf]

Table S2: The co-occurrence analysis parameters of microbial networks in the three groups

Attributes	Change	ETB	ETF
Vertex	73	68	96
Edge	793	159	1435
Average degree	21.73	4.68	29.90
Average path length	1.80	4.94	1.24
Network diameter	4	14	3
Clustering coefficient	0.79	0.57	0.68
Density	0.30	0.07	0.31
Heterogeneity	0.52	0.56	0.55
Centralization	0.23	0.08	0.31
Modularity	0.29	0.68	0.19
